# The roles of L1 transfer, L2 exposure, and morphological salience in bilingual children's L2 English morphological development

**DOI:** 10.3389/fpsyg.2025.1566442

**Published:** 2025-05-09

**Authors:** Xuan Wang, Stephanie McMillen, Yan Shi

**Affiliations:** ^1^Department of Linguistics, University of Kansas, Lawrence, KS, United States; ^2^Department of Communication Sciences and Disorders, College of Arts and Sciences, Syracuse University, Syracuse, NY, United States; ^3^Department of Linguistics, The University of Utah, Salt Lake City, UT, United States

**Keywords:** bilingualism, L2 morphological acquisition, L1 transfer, L2 exposure, morphological salience

## Abstract

**Introduction:**

The present study aims to advance our understanding of bilingual children's L2 morpheme acquisition variability by examining the child-internal factor of L1 transfer, child-external factor of L2 exposure, as well as the linguistic factor of morphological salience.

**Method:**

Drawing on naturalistic conversational data from a corpus of bilingual children's language samples, we analyzed the production accuracy of five English inflectional morphemes in 21 language samples from Spanish-English bilingual children and 25 language samples from Mandarin-English bilingual children. The two groups were age-matched and gender-balanced, with a mean age of 5 years and 1 month (range: 3; 8–7; 5). Binomial logistic mixed-effects models were employed to examine the accuracy of L2 morpheme production as influenced by L1 transfer, L2 input quantity (months of English exposure), input quality (morphological richness & lexical diversity), and the morphological salience of the target morphemes.

**Results:**

The results revealed a significant effect of L1 transfer, with Spanish-English bilinguals demonstrating higher accuracy than Mandarin-English bilinguals, particularly for English plural -s and articles. Besides, months of English exposure emerged as a significant positive predictor of L2 morpheme production accuracy. As regards input quality, while lexical diversity positively contributed to morpheme accuracy in both groups, morphological richness negatively affected morpheme production accuracy among Mandarin-English bilinguals. In addition, our descriptive analyses of morphological salience factors indicate that, across both groups of children, perceptual salience, morphophonological regularity, syntactic category and semantic complexity influence morpheme production accuracy to different degrees.

**Discussion:**

The study suggests that L1 transfer plays a critical role in L2 English morpheme production accuracy and underscores the importance of L2 input quantity and quality, such as lexical diversity, in explaining variability in bilingual children's morpheme acquisition. Additionally, difficulties in producing English morphemes may be associated with factors related to morphological salience. Overall, the findings underlie the importance of L1 transfer, L2 exposure, morphological salience in bilingual children's L2 morpheme acquisition.

## Introduction

The acquisition of morphemes in a second language (L2) by bilingual children has been intensively studied over the past few decades. Unlike monolingual children, bilingual children often demonstrate variability in L2 morpheme acquisition, which could be attributed to child-internal factors such as cross-linguistic transfer, cognitive abilities, aptitude as well as child-external factors such as language exposure (Bedore et al., 2018; Blom et al., 2010; Green, 1998; Michael and Gollan, 2005; Nicoladis, 2012; Paradis, 2010, 2011; among others). For instance, as a child-internal factor, cross-linguistic transfer from the first language (L1) is particularly important for L2 morpheme acquisition by bilingual children. Existing studies have revealed a positive transfer if both languages are typologically similar or a negative transfer if the L1 and L2 do not instantiate the same morphemes (e.g., Jia, 2003; Nicoladis et al., 2012, 2020; Paradis, 2011; Yip and Matthews, 2000). Besides, as a child-external factor, L2 exposure such as the cumulative input (e.g., the cumulative length of language exposure) and input quality (e.g., sources and linguistic richness of the input) has been attested to affect the acquisition of L2 morphemes by bilingual children (e.g., Bedore et al., 2012; Paradis, 2011; Unsworth, 2013).

In addition to child internal and external factors, linguistic factors are important for bilingual children's language acquisition (for an example in semantics, see McMillen et al., 2022). Morphological salience is a factor that is relevant for L2 morpheme acquisition, and in this study, it refers to the prominence and transparency of the form-meaning relationship of a given morpheme in the L2 (DeKeyser et al., 2018; Ellis, 2018; Goldschneider and DeKeyser, 2001). In the meta-analysis by Goldschneider and DeKeyser (2001), a set of morphological salience factors were shown to largely account for the morpheme acquisition order by adult and child L2 learners. These factors constitute salience at the phonological, morphological, syntactic, semantic, and frequency levels. However, no studies thus far have systematically examined morphological salience with bilingual children. Studying morphological salience is essential for understanding variability in bilingual children's L2 morpheme acquisition, as it is potentially interacting cross-linguistic transfer and language exposure. Salience of an L2 morpheme can facilitate positive transfer but lead to negative transfer when it lacks salience. To illustrate, plural morphemes are salient for Spanish-speaking learners of English, so they often have no difficulty using plural morphemes in English. In contrast, Mandarin-speaking learners, whose L1 lacks inflectional morphology, may struggle to notice or use plural morphemes in English. Salience is also related to language exposure, as bilingual children's exposure is distributed across two languages, resulting in potentially reduced frequency and variability for each language compared to monolingual learners. This unique input condition in bilingual children may amplify the role of morphological salience in morpheme acquisition more than for monolingual children.

The present study aims to advance the understanding of bilingual children's L2 morpheme acquisition variability by examining the child-internal factor of L1 transfer, child-external factor of L2 exposure, as well as the linguistic factor of morphological salience. Our analysis focuses on the production of five English morphemes in Spanish-English (SE) and Mandarin-English (ME) bilingual children, utilizing naturalistic conversation data drawn from the Child Language Data Exchange System (CHILDES; MacWhinney, 2000).

### L1 transfer in bilingual children's L2 English morpheme acquisition

Bilingual children are known to develop distinct yet interacting language systems, as per the interdependence hypothesis (Paradis and Genesee, 1996). The interdependence of the two languages explains how bilingual children's language systems influence one another through cross-linguistic interactions. Such interactions, often described as transfer, may result in bilingual children developing certain skills at a similar or different rate compared to monolingual peers (e.g., Jarvis and Pavlenko, 2008; Odlin, 1989).

In L2 morpheme acquisition, cross-linguistic transfer is often related to L1 morphological typology (e.g., Nicoladis, 2012; Nicoladis et al., 2012, 2020; Paradis, 2011; Yip and Matthews, 2000). Studies have shown that, bilingual children whose L1 is morphologically rich such as Spanish are likely to have a higher accuracy in morpheme production than bilingual children whose L1 is a morphologically isolating language such as Mandarin. For example, Paradis (2011) explored the acquisition of verbal morphology and vocabulary in L2 English by early sequential bilingual children aged 4; 10–7; 0 living in Canada whose family primarily used their L1 (e.g., Mandarin, Cantonese, Arabic, Hindi, Urdu, Punjabi, Spanish). In their results, L1 morphological system was one significant factor contributing to children's acquisition of L2 verbal morphology and vocabulary. Additionally, when dividing the children based on L1 typology, Paradis (2011) found that children whose L1 marks tense and agreement (Arabic, Hindi, Urdu, Punjabi, Spanish) have higher accuracy in English tense morphology than those whose L1 does not (Mandarin, Cantonese), suggesting that the L1 morphological typology plays an important role in L2 English morpheme acquisition by bilingual children.

In addition to morphological typology, cross-linguistic transfer can be triggered by specific L1 morphophonological properties. For instance, Nicoladis et al. (2012) examined tense-related morpheme production in sequential bilingual children (aged 5–12 yrs): French-English (L1 with tense marking) and Mandarin-English (L1 without tense marking). They analyzed accuracy and errors in regular and irregular verbs. For example, they coded using bare verbs as stem errors (e.g., *He bring the cake*), irregular verbs with regular morphemes as overregularization errors (e.g., *He bringed the cake*), and irregular verbs with incorrect irregular forms as irregularization errors (e.g., *He brang the cake*). Nicoladis et al observed an L1 transfer effect: Mandarin-English bilinguals performed better on irregular verbs due to their L1 preference for monosyllabic words and internal tense marking, while French-English bilinguals showed higher accuracy for regular verbs, reflecting similar tense morphology in French and English. Additionally, Mandarin-English children frequently used bare verb stems instead of overregularization, which could be explained by the fact that Mandarin does not mark tense overtly. Instead, Mandarin includes bare verbs with temporal phrases to express tense and aspects, thus leading to omission errors in their L2 English morpheme production.

Building on Nicoladis et al. (2012) and Nicoladis et al. (2020) extended the investigation to younger bilingual children to explore the source of transfer in tense morpheme acquisition. They tested 27 Mandarin-English bilinguals aged 4; 0–6; 7, who were sequential bilinguals exposed to Mandarin from birth and began learning English between ages 2 and 5. Participants' morpheme production accuracy was assessed with a language sample retell task. Consistent with previous findings, their study identified a higher accuracy for irregular forms than regular forms. Nicoladis et al. attributed this finding to morphophonological transfer because these children attempted to avoid complex codas such as adding *-ed* to verbs (e.g., *hop* vs. *hopped*), as complex codas are not allowed in Mandarin (Duanmu, 2007).

In summary, studies have shown that L1 morphological typology (e.g., fusional or isolating) as well as the feature of morphophonology of the L1 (e.g., Mandarin preferring internal changes instead of adding a coda) influence the L2 morpheme production accuracy of bilingual children, highlighting the important role of cross-linguistic L1 transfer in L2 morpheme production.

### L2 exposure in bilingual children's L2 English morpheme acquisition

Extensive research has examined the relationship between language exposure and language outcomes in bilingual children. Findings consistently indicate that more advanced language outcomes are often associated with greater input of a given language (e.g., Bedore et al., 2012; Bohman et al., 2010; Jia and Fuse, 2007; Paradis, 2023, among others). For example, Jia and Fuse (2007) examined the acquisition of English grammatical morphemes in a study involving 10 Mandarin-speaking children learning English in the U.S. Their findings revealed a general positive correlation between the length of English immersion and the children's production of target structures in English. Bedore et al. (2012) investigated how first and current language exposure impacts morphosyntactic and semantic development in Spanish-English bilingual children, and they found that the current exposure to English and Spanish significantly predicts the language task outcomes more than the age of first exposure, highlighting the important role of input quantity in bilingual children's language acquisition.

In addition to quantity, high quality adult-children interactions are associated with children's language development such as vocabulary learning, literacy, and academic achievement (e.g., Paradis, 2011). Input quality can be assessed across various dimensions, such as the richness of L2 exposure at home, the sources of interaction, engagement in language activities, linguistic complexity, etc. (see Rowe and Snow, 2020 for a review). For example, Paradis (2011) evaluated input quality by examining the frequency of children's participation in activities such as reading, TV watching, storytelling, and singing. Alongside other child-internal and external factors, Paradis (2011) found that both input quality and quantity significantly explained variance in L2 vocabulary and verbal morphology, with input quality accounting for a larger proportion of the variance than input quantity. Similarly, Place and Hoff (2011) conducted a diary study with Spanish-English bilingual children to assess the impact of language input quantity and quality on their vocabulary and grammatical development, as measured by the MacArthur-Bates Inventories (Fenson et al., 2003; Jackson-Maldonado et al., 2003). They found that input quality—specifically, the number of different English speakers in the input source and the proportion of native English input—had a more significant effect on English vocabulary and grammar learning than input quantity. This finding is consistent with evidence suggesting that phonological, lexical, and syntactic variability and diversity facilitate language development (Fisher et al., 2004; Richtsmeier et al., 2009).

However, most existing studies focused on the input from caregivers. Of course, this is not the only language input source of children. Children also interact with other interlocutors such as the siblings, teachers, and sometimes experimenters, etc. There are few studies exploring how input quality of interlocutors such as teachers or experimenters influences bilingual children's language development. Bowers and Vasilyeva (2011) is one study that examined child L2 learners' vocabulary learning in relation to the teachers' input quality measured by mean lengths of utterance (MLU) and lexical diversity. While no significant effect was revealed for lexical diversity of teachers' input, MLU was found to negatively affect children's vocabulary growth. The authors attributed this inverse relationship to children's difficulty in deconstructing morphemes from teachers' input for word learning at early stage of learning. Another study by Sun et al. (2018) investigated the vocabulary and receptive grammar learning of Chinese-English bilinguals, considering both child-internal and child-external factors, such as language exposure, school English input, and home English media input. Sun et al. found that the amount of school input was the strongest predictor of receptive grammar, while home media input significantly influenced both productive and receptive vocabulary. These findings highlight the importance of teachers' input in bilingual children's L2 acquisition.

Taken together, language exposure plays a crucial role in bilingual children's L2 development. Studies have identified significant positive relationships between both input quantity and input quality, and bilingual children's L2 acquisition. However, different measures of input quality (e.g., MLU vs. lexical diversity) may influence language development in different ways (Bowers and Vasilyeva, 2011). Moreover, most research on input quality has focused on its effects on vocabulary learning; while some studies have explored grammar learning, they typically address grammar in a general sense rather than specifically targeting morphological learning. Consequently, the impact of input quality on bilingual children's L2 morpheme acquisition remains unclear. Additionally, few studies have examined the effects of interactive input quality from non-caregivers on L2 morpheme acquisition in bilingual children. Investigating the effects of input quality, such as lexical diversity and MLU, is essential to further our understanding of how interactive input influences bilingual children's L2 morpheme development.

### Morphological salience in bilingual children's L2 English morpheme acquisition

In the study of salience, some researchers (e.g., DeKeyser et al., 2018; Ellis, 2018; Goldschneider and DeKeyser, 2001) used a “narrow” view and defined salience in perceptual and linguistic aspects, whereas others (e.g., Leow and Martin, 2017) focused a “wide” view and defined salience from a discourse context perspective. The current study adopted the “narrow” view, defining morphological salience as being perceptually sonorous with a transparent linguistic form-meaning relationship (Goldschneider and DeKeyser, 2001). Specifically, morphological salience is operationalized as five factors: perceptual salience, semantic complexity, morphophonological regularity, syntactic category, and frequency.

Perceptual salience refers to how easily one can perceive a morpheme based on acoustic features. A morpheme with higher perceptual salience will be acquired earlier than those with relatively lower perceptual salience. For example, the free morpheme *at* contains the open low front vowel/æ/which is sonorous and salient (Laver, 1994), resulting in relatively early acquisition of this morpheme. Semantic complexity indicates how many meanings a given form can convey. A morpheme that has fewer meanings will be easier to acquire due to transparency between the form and meaning compared to morphemes with more meanings. For example, an article conveys one meaning—the definiteness (e.g., an apple indicates one general apple)—while third-person singular *-s* conveys three meanings: the present tense, the person, and the number (e.g., *He eats an apple* indicates that the “eating apple” action is performed in present moment by one person in a third-person view). Morphophonological regularity refers to the extent that a morpheme is influenced by the phonological context. The more phonologically regular a morpheme is, the earlier it will be acquired. For instance, third-person singular *-s* has three phonological allophones (/s/, /z/, /z/) and with homophony to the plural *-s*, so it may be relatively more difficult to acquire. Syntactic category is related to the Functional Category theory by Zobl and Liceras (1994) where a lexical morpheme is easier to acquire than a functional morpheme, and a free morpheme is easier to acquire than a bound morpheme. Frequency is the last factor in the morphological salience construct and comprises the number of times a morpheme was presented to the listener. The more frequently one hears a target morpheme, the easier or earlier it will be acquired.

Salience is important for bilingual children who are susceptible to L1 transfer and whose language input is inherently divided between their two languages. Research suggests that differences in exposure can influence the acquisition of linguistic properties depending on their opacity or complexity (Blom et al., 2012; Gathercole, 2002; Paradis, 2010), but due to bilingual children having to split their time between two languages, the impact of these factors may be stronger for bilingual children than monolingual children (Unsworth, 2016). In this sense, morphological salience contributes to bilingual children's detection of target forms within the input. For example, in a meta-analysis study, Goldschneider and DeKeyser (2001) investigated how these salience factors affect the acquisition order of six English morphemes (plural *-s*, possessive *-'s*, third-person singular *-s*, articles, past tense *-ed*, and progressive *-ing*). Their analysis, covering 12 studies (published from 1973 to 1996) with child and adult L2 English learners, revealed that these salience factors explained significant variance in morpheme production accuracy. In empirical studies, salience has been identified as a critical factor in second language acquisition (Gass et al., 2017). For instance, Whittle and Lyster (2016) investigated the acquisition of Italian subject-verb agreement by Chinese-speaking children. Given the absence of agreement marking in Chinese, subject-verb agreement was argued to lack salience for these learners. Nevertheless, targeted interventions emphasizing the forms led to improved accuracy, highlighting the role of salient input. Similarly, Schwartz et al. (2016) examined morphological awareness in Arabic-Hebrew and Hebrew-Arabic bilingual children, and identified evidence of cross-linguistic transfer, particularly with the Arabic bound possessives and dual number, which resulted in high accuracy on Hebrew morphological tests. They attributed these findings to the salience of these two morphemes in Arabic.

Despite its importance, research on the role of salience in bilingual children's L2 morpheme acquisition remains scarce. Most existing studies primarily focused on one or two aspects of salience, rather than systematically examining a range of salience factors. Even though Goldschneider and DeKeyser (2001) conducted a meta-analysis with a large number of studies, both children and adults were included in their analysis, and so, the specific impact of salience on young bilingual children's English acquisition could not be isolated. Moreover, their statistical methods have been criticized for inaccuracies in estimating predictive power (see Murakami, 2014 for further discussion). Furthermore, some studies, such as O'Grady et al. (2017), offer an alternative perspective on L2 morpheme acquisition. They challenge the salience-based explanation, arguing that the difficulty in acquiring inflectional morphemes such as *-s* in the L2 is not due to their acoustic salience, but rather because L2 learners face heavy cognitive loads and processing inefficiencies in acquiring L2 morphemes. It is important to note that O'Grady et al. (2017) primarily focused on adult L2 learners, and in their study, salience was operationalized as perceptual acoustic salience, without considering other salience factors. Therefore, to better understand the role of morphological salience, a comprehensive investigation of various salience factors is needed to provide valuable insights into the sources of variability in bilingual children's L2 morpheme acquisition.

### The current study

The current study examines bilingual children's L2 English morpheme production, focusing on L1 typology, L2 exposure, and morphological salience. We investigate the production of five English morphemes^1^ (i.e., plural *-s*, third-person singular *-s*, articles, regular past tense *-ed*, present progressive *-ing*) by Spanish-English (SE) bilinguals and Mandarin-English (ME) bilinguals. The typological differences between Spanish and Mandarin allow us to evaluate the effects of L1 transfer on L2 morpheme acquisition. We also aim to address how L2 exposure, especially different measures of input quality (e.g., lexical diversity and morphological richness) affect L2 morpheme acquisition by bilingual children. Furthermore, the current study aims to conduct an exploratory analysis on the extent morphological salience factors influence bilingual children's L2 English morpheme production. While previous studies have offered valuable insights into the role of salience in L2 acquisition, they have not systematically examined a range of salience factors with bilingual children. There is also a debate regarding the extent to which variability in L2 morpheme acquisition can be explained by morphological salience. Our study addresses this gap by investigating a comprehensive set of salience factors. Addressing this inquiry will help understand how morphological salience factors interact with child internal (i.e., L1 typology) and external factors (i.e., L2 exposure) in bilingual children's L2 morpheme production. Specifically, we seek to address the following research questions.

#### RQ1: is there evidence of cross-linguistic influence where the L1 (Spanish vs. Mandarin) impacts bilingual children's L2 English morpheme production accuracy?

##### Predictions

In line with previous studies (e.g., Paradis, 2011; Nicoladis et al., 2012, 2020), SE bilinguals are predicted to have an overall higher accuracy for morpheme production compared to ME bilingual children due to the different L1 morphological typologies. Besides, studies revealed positive L1 transfer effect when there is a corresponding morpheme between L1 and L2, so we expect to see cross-linguistic facilitation effects for bilingual children with corresponding morphemes but not with non-corresponding morphemes. Mandarin Chinese does not encode articles, third-person singular -*s*, and past tense -*ed*, plural -*s* in the linguistic system. Thus, we predict that SE bilinguals have higher accuracy compared to ME bilinguals for these morphemes. Note that we do not predict a group difference in the present progressive *-ing*, as although Chinese does not have the corresponding *-ing* morpheme, it has aspect markers such as the pre-verbal *zai* or post-verbal *zhe*, which may facilitate their acquisition of present progressive *-ing*. Also note Chinese uses the lexical item “-*men*” to pluralize some nouns, but “-*men*” is argued to be different from English plural -*s* because it is limited in use in Mandarin (Li, 1999; Corbett, 2000; Zhang, 2008). Thus, we expect no facilitation effect from lexical item “-*men*” on plural -*s* acquisition for ME bilingual children.

#### RQ2: to what extent does L2 exposure (cumulative input quantity and input quality) affect the accuracy of L2 English morpheme production by bilingual children?

##### Predictions

Consistent with prior research (e.g., Bedore et al., 2012; Unsworth, 2013), we predict that children with higher cumulative input quantity will demonstrate greater accuracy in producing English morphemes. Building on Paradis (2011), we anticipate that input quality of lexical diversity and morphological richness would serve as a predictor of morpheme production accuracy. However, the effects of lexical diversity and morphological richness on vocabulary growth have been mixed in prior research (e.g., Bowers and Vasilyeva, 2011), leaving predictions for L2 morpheme production accuracy open to interpretation. It is possible that lexical diversity will exhibit significant or non-significant effects, while morphological richness may potentially exert a negative effect on morpheme production accuracy.

#### RQ3: how do morphological salience factors influence the accuracy of L2 English morpheme production by bilingual children?

##### Predictions

Morphological salience is predicted to affect L2 morpheme acquisition (e.g., Goldschneider and DeKeyser, 2001). For both groups of bilingual children, we predict that a high degree of perceptual salience would lead to high accuracy, which is in line with studies by Whittle and Lyster (2016) and Schwartz et al. (2016). However, it is also possible that perceptual salience may not play a significant role, as argued by O'Grady et al. (2017). In addition, semantic complexity and morphophonological irregularity may result in low accuracy, due to the opaque form-meaning relationship. As for syntactic category, a morpheme with low values (bound and functional) would have lower accuracy than a morpheme with high values (free and lexical), as studies have shown that children tend to have more difficulties with inflectional morphemes than lexical morphemes (e.g., Zobl and Liceras, 1994).

## Method

### Corpus and participants

The present study retrieved language samples from Paradis's (2005) corpus in the bilingual collection of the CHILDES database (MacWhinney, 2000). Paradis's (2005) corpus consists of longitudinal and naturalistic conversation data between sequential bilingual children (mean age = 5; 6) and the experimenters. The language samples were from 25 children of various L1s who were learning English as their second language after immigrating to Canada with their families. Data were collected in five time points over a two-year span, with ~6 months between each point in time. During the conversation, children were asked a series of questions by the experimenters to elicit target English morphemes, such as “*Did you/Are you going to have a birthday party?*”, “*What happens at a birthday party*?”, etc. (Paradis, 2005).

Among the child participants in the corpus, children whose L1 was Mandarin or Spanish were selected as two groups of interest in our study because of the differences in L1 morphological typology. To make sure the two groups of children were homogeneous with matched age and similar English proficiency, three children were removed in our analyses, resulting in 25 transcripts from five Mandarin-English (ME) bilingual children and 21 from six Spanish-English (SE) bilingual children. The demographic information is provided in Table 1 and Appendix A. Age of English acquisition (AOE), the Age at testing (AAT), and mean length of utterances in morphemes (MLUm)^2^ are comparable between the two groups of bilingual children.

**Table 1:** Participant information.

**Group**	**Statistic**	**AOE**	**AGE**	**MOE**	**MLUm**
SE bilinguals (*n* samples = 21)	Mean	60.52	79.52	19.00	3.93
	SD	12.12	11.39	9.70	0.95
	Range	46–89	61–100	5–40	2.05–5.39
ME bilinguals (*n* samples = 25)	Mean	59.72	79.28	19.56	4.09
	SD	9.31	12.61	9.72	0.74
	Range	47–73	54–103	4–42	2.79–5.41

AOE, age of English exposure; AGE, age at testing (months); MOE, months of English exposure; MLUm, mean lengths of utterances in morphemes.

### The independent measures

In this study, we included language group, L2 exposure, and morphological salience as independent factors. Language group is a two-level categorical factor (Spanish = −0.5, Mandarin = 0.5). L2 exposure was operationalized using Months of English Exposure (MOE) as a measure of input quantity, and MLUm and lexical diversity D (Malvern et al., 2004) as measures of input quality. MLUm is as a measure of morphological richness of the input, which is determined by the number of morphemes over a sample of utterances (Brown, 1973). The D measure is defined as “the range and variety of vocabulary” (McCarthy and Jarvis, 2007, p. 459) and is used as an index of lexical diversity. All continuous factors were centered from their means. In addition, we also controlled for morpheme types which were sum-coded in five levels corresponding to the five target morphemes. Morphological salience values for each morpheme were coded following the protocols outlined by Goldschneider and DeKeyser (2001). A detailed description of the coding for each factor is provided below and in Appendix B. Note that, unlike Goldschneider and DeKeyser (2001), we excluded the frequency factor from our analyses, as their frequency data were based on monolingual English parental input reported in Brown (1973), which does not accurately reflect the input frequency for bilingual children who are learning English as an L2.

#### Perceptual salience

For perceptual salience, three subfactors (number of phones, syllabicity, sonority) were coded and added together to comprise one combined factor for each morpheme. We calculated the number of phones in all allomorphs of a given morpheme. For instance, the third-person singular *-s* has three allomorphs ([s], [z], [z]) and four distinct phones in total. This results in an average of 1.33 phones per allomorph (4 total phones/3 allomorphs = 1.33). Syllabicity and sonority are two additional perceptual salience factors, measuring whether a morpheme contains a vowel and how sonorous each phone is within the morpheme, based on the sonority hierarchy by Laver (1994). To illustrate, a low vowel has the largest sonorous value (9 being the maximum) and a stop consonant has the smallest sonorous value (1 being the minimum). Thus, the morpheme “at” [æt] is assigned the value 11 because it receives one point for containing one vowel, nine points for a low vowel [æ], and one point for the stop consonant [t] (1 + 9 + 1 = 11).

#### Morphophonological regularity

Morphophonological regularity is comprised of two subfactors: phonological alternations and morphological homophony. First, we coded the number of phonological alternations for each morpheme. For instance, regular past tense *-ed* contains three alternations (i.e., [t, d, d]), so regular past tense *-ed* will be assigned a value of 3. Additionally, we calculated whether a given morpheme has homophony (no homophony = 1, homophony = 2). For example, plural *-s* has three phonological alternations and it is homophonous with possessive *-'s* and third-person singular *-s*; thus, plural *-s* is assigned 4 for its morphophonological regularity.

#### Semantic complexity

Semantic complexity is coded based on how many meanings can be expressed by a specific morpheme. For instance, the article is assigned one point because it is used for definiteness, while third-person singular *-s* is assigned three points because it can be used to indicate present tense, person, and number.

#### Syntactic category

Regarding the syntactic category, our coding is based on the Functional Category Theory by Zobl and Liceras (1994; see also Goldschneider and DeKeyser, 2001 for additional details). According to the Functional Category Theory, a lexical morpheme is typically acquired earlier than a functional morpheme, and a free morpheme is typically acquired before a bound morpheme. For example, a free lexical morpheme, such as the preposition “on,” receives the highest possible point value (4 points) while a bound functional morpheme, such as present progressive *-ing*, receives the lowest point value (1 point).

### The dependent measure

Previous research on language sample analysis recommends using at least 50–100 utterances or a 7–10-min language sample to obtain a representative and reliable measure of a child's language use (e.g., Guo and Eisenberg, 2015). Following this recommendation, we analyzed the first 200 intelligible utterances for each child to ensure a representative language sample at each time point. The inclusion of 200 intelligible utterance exceeds the minimum requirement suggested by Guo and Eisenberg (2015), further enhancing the representativeness of the language samples. Within these 200 utterances, we evaluated five target English morphemes that were tested for salience effects in Goldschneider and DeKeyser (2001). Initially, we included the possessive -'*s* in our analysis; however, due to the limited number of obligatory contexts of this morpheme, we excluded it from our analyses. For the remaining five morphemes, coding included: (a) the number of correct child production of the target morpheme, (b) the number of obligatory contexts in the child's utterances, and (c) the number of child production in non-obligatory contexts. We assigned no scores to a morpheme if it appeared in fewer than four obligatory contexts, as such cases do not provide reliable information about the child's use of that morpheme.

Target-Like Use (TLU) is adopted as a measure of accuracy, as it accounts for the developmental errors, overgeneralization, and overuse of correct morphemes in children's production. TLU is obtained by dividing the number of correct suppliance in obligatory contexts over the number of obligatory contexts plus the number of suppliance in non-obligatory contexts. This formula from Goldschneider and DeKeyser (2001, p. 25) is reproduced below.


(1) $$\begin{array}{l} \text{TLU=}\\ \frac{\#\text{of correct production}/\text{suppliance in obligatory contexts}}{\#\text{of obligatory contexts +}\;\#\text{of production}/\text{suppliance in non}-\text{obligatory contexts}} \end{array}$$

The data coding was first completed independently by the first and third authors of this study, and the preliminary coding results were compared and discussed to ensure consistency. Then, a trained student research assistant coded three transcripts from SE and three from ME bilingual children. The inter-rater reliability for the coding of these six transcripts was calculated, yielding an average Cronbach's alpha value of 0.973, indicating high consistency between coders.

### Data analysis

Data were analyzed using the *lme4* package (Bates et al., 2014) for binomial logistic mixed-effects models with the *lmerTest* package for *p*-values (Kuznetsova et al., 2016). In our models, we transformed the TLU percentage scores as TLU_Correct and TLU_Incorrect to account for the bounded nature of the TLU, ensuring that predictions remain within the valid range and appropriately handling the proportional nature of TLU scores.

For the first two research questions which examine the effects of L1 transfer and L2 exposure, the fixed effects included Language Group (SE vs. ME), Months of English Exposure (MOE), the MLU by the experimenter (EXP_MLU), Lexical D, and their interactions, controlled by Age and Morpheme types. A by-subject random intercept was incorporated to account for individual variability. The model fit was assessed with the DHARMa package (Hartig, 2024), which showed no issues with dispersion or outliers. Besides, multicollinearity was assessed by calculating the Generalized Variance Inflation Factor (GVIF) using the *car* package in R (Fox and Weisberg, 2018), and the values are well within the acceptable limits.

For the third research question concerning morphological salience, we employed a descriptive rather than an inferential analysis for the following reason. To illustrate, the salience predictors exhibit only five unique combinations of values corresponding to the five target morphemes. Thus, the values of these predictors are invariant for each morpheme, resulting in a model that is conceptually similar to a saturated design with four predictors for five observations. This issue was pointed out by Murakami (2014) for the approach used by Goldschneider and DeKeyser;s (2001), where the limited variability across predictors at the morpheme level leads to overfitting and unreliable results. Given the fundamental limitation, conducting inferential analyses is inappropriate. Instead, using a descriptive approach enables us to explore the potential relationship between the morphological salience and morpheme-level performance without overfitting the data.

## Results

### The role of L1 transfer in L2 morpheme acquisition

The descriptive TLU scores of the five target morphemes are presented in Table 2. As we can see, SE bilinguals scored higher than ME bilinguals on articles (91% vs. 83%) and plural -s (92% vs. 84%). In contrast, ME bilingual children outperformed SE bilingual children in producing past tense *-ed* (85% vs. 78%). Both groups performed similarly for present progressive *-ing*. Notably, both groups showed the lowest accuracy scores in producing the third-person singular *-s* morpheme, with 63% for SE bilingual children and 50% for ME bilingual children.

**Table 2:** Descriptive TLU scores for each morpheme between language groups.

**Morpheme**	**SE bilinguals Mean (SD)**	**ME bilinguals Mean (SD)**
Articles	91% (8)	83% (13)
Plural *-s*	92% (6)	84% (13)
Past tense *-ed*	78% (27)	85% (21)
PP *-ing*	82% (21)	78% (26)
3SIG *-s*	63% (38)	50% (33)

Focusing on the effect of L1 transfer, Model 1 (presented in Table 3) revealed a significant main effect for Group (*Est*. = 0.46, *SE* = 0.20, *t* = 2.26, *p* = 0.023), indicating a significant difference in production accuracy between ME and SE bilingual children. Moreover, the significant positive effects of morpheme level 1 (Plural-*s*; *Est*. = 0.538, *p* < 0.001) and morpheme level 4 (Articles; *Est*. = 0.61, *p* < 0.001) suggest that both groups of children produced these two morphemes with higher accuracy than other morphemes. The third-person singular *-s* shows the largest negative deviation from the mean (−0.53 – 0.11 + 0.10 – 0.61 = −1.15), indicating that children had difficulty producing it. In addition, separate binominal logistic mixed-effects models were performed, which revealed a significant group difference in plural *-s* and articles but not on other morphemes. For plural *-s*, SE bilinguals were significantly more accurate than ME bilinguals [SE: 92% vs. ME: 84%, *F*_(1,44)_ = 6.19, *p* = 0.01, η*2*_*G*_ = 0.12]. Similarly, SE bilinguals produced articles with higher accuracy than ME bilinguals [SE: 91% vs. ME: 83%, *F*_(1,44)_ = 5.62, *p* = 0.02, η*2*_*G*_ = 0.11]. No significant group differences were found for the other morphemes.

**Table 3:** Model 1: TLU as a function of language group, input quantity and quality.

**Predictor**	**Estimate**	**Std. error**	***z*-value**	**Pr(>|*z*|)**	**Significance**
(Intercept)	1.69076	0.10989	15.385	< 2e-16	^***^
Group	0.46685	0.20657	2.26	2.38e-02	^*^
MOE	0.57563	0.12252	4.698	2.63e-06	^***^
EXP_MLU	−0.43491	0.24079	−1.806	7.09e-02	.
D	0.08783	0.02444	3.594	0.000325	^***^
Morpheme 1	0.53889	0.1001	5.383	7.31e-08	^***^
Morpheme 2	0.11701	0.15857	0.738	4.61e-01	
Morpheme 3	−0.10249	0.18395	−0.557	0.577423	
Morpheme 4	0.61697	0.09326	6.616	3.69e-11	^***^
Age	−0.23149	0.11476	−2.017	0.043691	^*^
Group: MOE	−0.32213	0.23064	−1.397	0.162516	
Group: EXP_MLU	0.01511	0.46478	0.033	0.974063	
MOE: EXP_MLU	0.25816	0.29201	0.884	0.376656	
Group: D	0.11835	0.04471	2.647	0.008118	^**^
MOE: D	0.01058	0.02214	0.478	0.63297	
EXP_MLU: D	−0.12335	0.04704	−2.622	0.008739	^**^
Group: MOE: EXP_MLU	−1.04782	0.60108	−1.743	0.081295	.
Group: MOE: D	0.06992	0.04524	1.546	0.122214	
Group: EXP_MLU: D	0.3049	0.09335	3.266	0.001091	^**^
MOE: EXP_MLU: D	0.19113	0.07199	2.655	0.007936	^**^
Group: MOE: EXP_MLU: D	0.35594	0.143	2.489	0.012805	^*^

MOE, months of English exposure; EXP_MLU, experimenter MLU; D, lexical diversity.

^***^p ≤ 0.001, ^**^p ≤ 0.01, ^*^p ≤ 0.05, ^.^p ≤ 0.1.

In sum, we observed a significant group difference in the production of the English morphemes (esp. plural *-s* and articles), with SE bilinguals achieving higher TLU scores than the ME bilinguals, which indicates the presence of an L1 transfer effect.

### The role of L2 exposure in L2 morpheme acquisition

We analyzed the factors of L2 exposure including input quantity (MOE), and the interactive input quality of the experimenter (D and EXP_MLU). As seen in Table 3, a significant main effect of MOE (*Est*. = 0.57, *SE* = 0.12, *t* = 4.69, *p* < 0.001) suggests that the quantity of English exposure plays a significant role in bilingual children's morpheme production accuracy. No significant interaction between Group and MOE (*p* = 0.16) was found, suggesting that for both bilingual groups, children with greater English exposure produced the target English morpheme more accurately than those with less English exposure.

Regarding the effect of input quality, an emerging trend was observed for EXP_MLU (*Est*. = −0.43, *SE* = 0.24, *t* = −1.806, *p* = 0.07), suggesting a potential negative influence of the experimenter's MLU on children's morpheme production accuracy. In contrast, the lexical D was a significant predictor of morpheme production accuracy (*Est*. = 0.08, *SE* = 0.02, *t* = 3.59, *p* < 0.001). These findings indicate that input quality of the experimenter, particularly lexical diversity plays a crucial role, whereas morphological richness may have a negative impact.

It is important to note that in Model 1 we observed a significant 4-way interaction between Group, MOE, EXP_MLU and D, which motivates us to conduct separate group analyses to understand the nature of this interaction. As seen in Table 4, Model 2 (SE bilingual children) indicated a marginally significant effect of MOE on morpheme production accuracy (*p* = 0.051). Although the experimenters' MLU was not a significant predictor, the lexical diversity of input emerged as a significant predictor of morpheme production accuracy for SE bilingual children. In comparison, Model 3 (ME bilingual children) revealed a significant positive effect of MOE, with more English exposure associated with higher accuracy. Besides, the experimenters' MLU was shown to have a negative effect on the accuracy (*Est*. = −0.74, *SE* = 0.30, *t* = −2.469, *p* = 0.013), whereas the lexical diversity is associated with higher production accuracy (*Est*. = 0.06, *SE* = 0.02, *t* = 2.38, *p* = 0.017).

**Table 4:** **(A)** Model 2: TLU as a function of input quantity and quality for SE bilinguals. **(B)** Model 3: TLU as a function of input quantity and quality for ME bilinguals.

**Predictors**	**Estimate**	**Std. error**	***z*-value**	**Pr(>|*z*|)**	**Significance**
**A: SE bilinguals**					
(Intercept)	2.04216	0.17406	11.733	< 2e-16	^***^
MOE	0.32989	0.16965	1.945	5.18e-02	.
EXP_MLU	−0.23501	0.28816	−0.816	4.15e-01	
D	0.11041	0.03383	3.264	1.10e-03	^**^
Morpheme 1	0.78821	0.17234	4.574	4.79e-06	^***^
Morpheme 2	−0.10252	0.22731	−0.451	6.52e-01	
Morpheme 3	−0.21061	0.2934	−0.718	4.73e-01	
Morpheme 4	0.73932	0.1552	4.764	1.90e-06	^***^
Age	0.3364	0.18576	1.811	7.01e-02	.
MOE: EXP_MLU	0.58765	0.46277	1.27	0.204137	
MOE: D	0.02132	0.02763	0.772	0.440391	
EXP_MLU: D	0.00139	0.05153	0.027	9.78e-01	
MOE: EXP_MLU: D	0.33154	0.09578	3.462	0.000537	^***^
**B: ME bilinguals**					
(Intercept)	1.54116	0.11871	12.982	< 2e-16	^***^
MOE	0.94214	0.13012	7.24	4.48e-13	^***^
EXP_MLU	−0.74744	0.30272	−2.469	1.35e-02	^*^
D	0.06133	0.02576	2.381	1.73e-02	^*^
Morpheme 1	0.40221	0.12526	3.211	0.00132	^**^
Morpheme 2	0.35347	0.22247	1.589	1.12e-01	
Morpheme 3	−0.06499	0.236	−0.275	7.83e-01	
Morpheme 4	0.52501	0.11781	4.456	8.34e-06	^***^
Age	−0.55951	0.1159	−4.827	1.38e-06	^***^
MOE: EXP_MLU	0.88554	0.34318	2.58	0.00987	^**^
MOE: D	−0.03783	0.02696	−1.403	0.16051	
EXP_MLU: D	−0.27639	0.05917	−4.671	2.99e-06	^***^
MOE: EXP_MLU: D	0.03033	0.08103	0.374	0.70816	

MOE, months of English exposure; EXP_MLU, experimenter MLU; D, lexical diversity.

^***^p ≤ 0.001, ^**^p ≤ 0.01, ^*^p ≤ 0.05, ^.^p ≤ 0.1.

Together, these findings underscore the critical role of L2 exposure, with increased English input facilitating more accurate morpheme production in both groups of bilingual children. Regarding input quality, lexical diversity in the input was associated with greater production accuracy for both groups. Notably, experimenters' input morphological richness had a negative effect on production accuracy for ME bilingual children, but it has no significant main effect for SE bilingual children.

### The role of morphological salience in L2 morpheme acquisition

**Figure 1:**
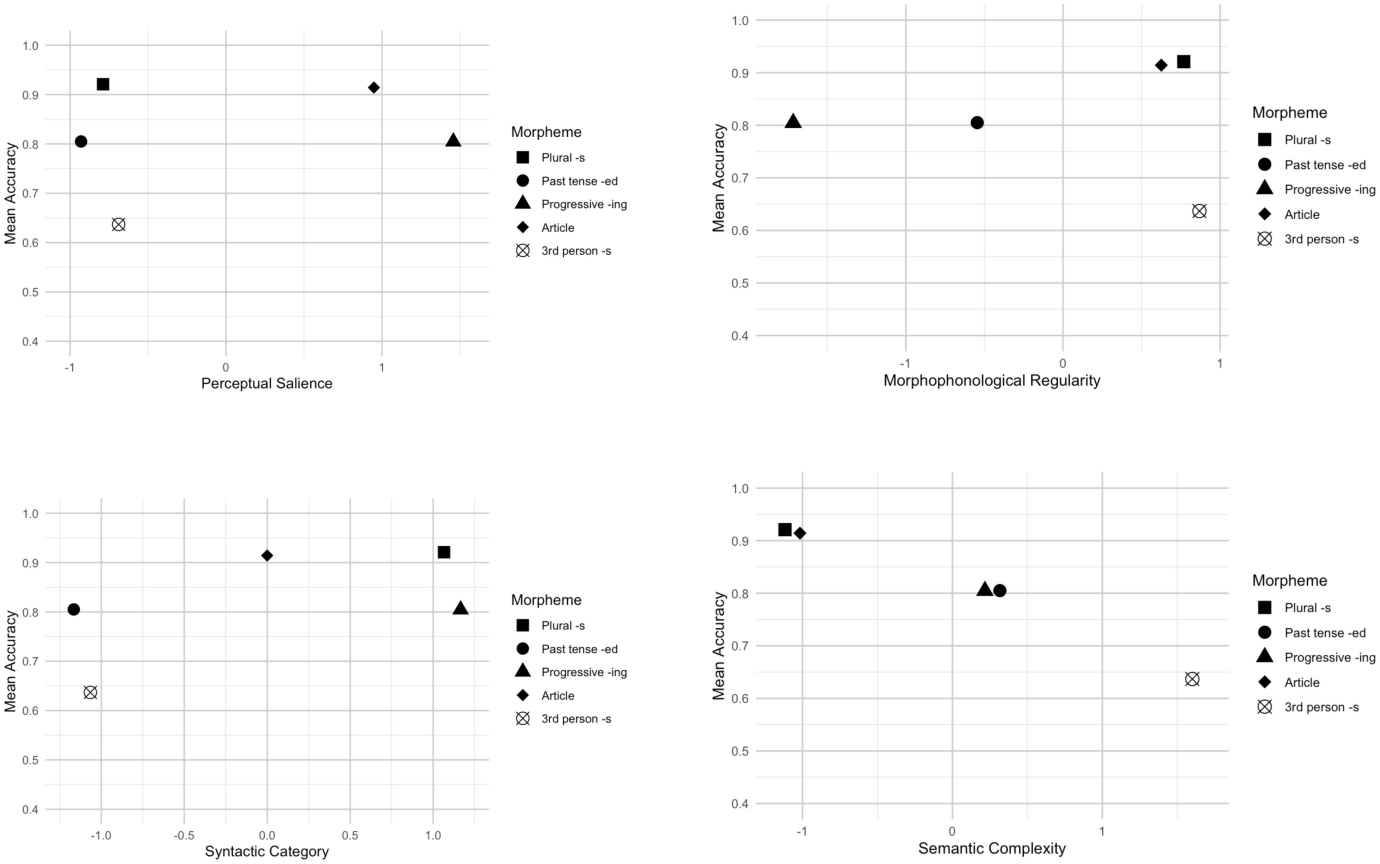
Morphological salience and morpheme production accuracy (Spanish–English bilinguals).

**Figure 2:**
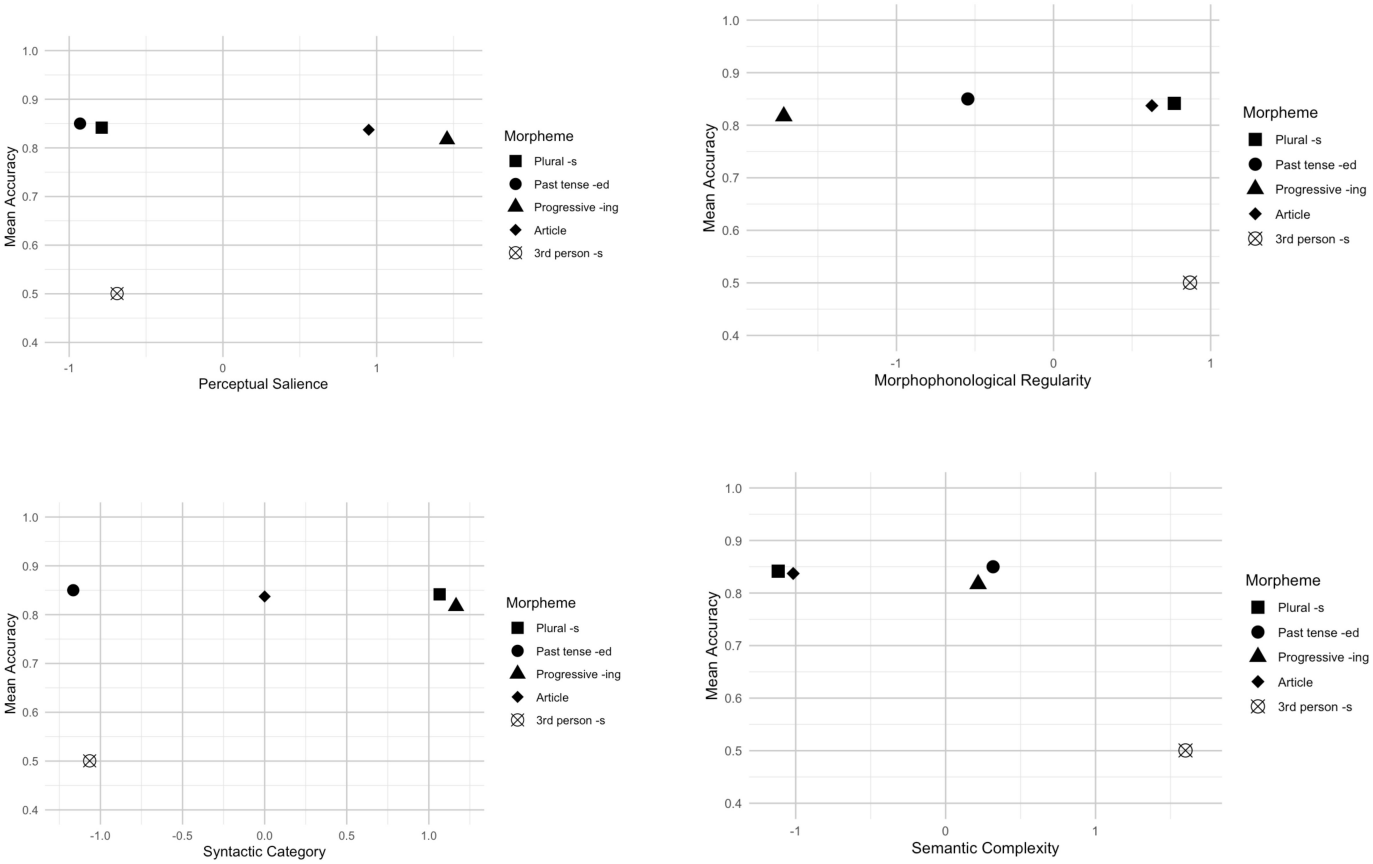
Morphological salience and morpheme production accuracy (Mandarin–English bilinguals).

We adopted an exploratory approach to investigate the role of morphological salience in bilingual children's acquisition of L2 English morphemes. To this end, we plotted morpheme production accuracy across four morphological salience factors in Figures 1, 2.

SE bilingual children's results were presented in Figure 1. In terms of perceptual salience, the third-person singular *-s* (3sig) with low perceptual salience showed lower accuracy whereas articles and the present progressive -*ing* with high perceptual salience showed higher accuracy. However, despite their low perceptual salience scores, the production of plural *-s* and past tense *-ed* was highly accurate. As regards morphophonological regularity, although accuracy seems to decrease with increased irregularity, exceptions were observed, such as the present progressive *-ing*, articles, and plural *-s*, which demonstrated high accuracy regardless of their morphophonological regularity. In terms of syntactic category, a general gradient effect was observed, with plural *-s* and articles showing higher accuracy than the third-person singular -s. Semantic complexity, on the other hand, exhibited an inverse relationship with morpheme production accuracy, such that accuracy declined as complexity increased.

As shown in Figure 2 for ME bilingual children, the present progressive -*ing* and articles, with high perceptual salience, exhibited high production accuracy, whereas the third-person singular *-s*, which has low perceptual salience, demonstrated low accuracy. However, the past tense *-ed* and plural *-s* also demonstrated high accuracy despite their low perceptual salience. Regarding morphophonological regularity, each morpheme's production accuracy appeared to be distributed across different levels of regularity. For instance, the third-person singular *-s* which has greater irregularity showed lowest accuracy, however, other morphemes exhibited high accuracy regardless of their regularity levels. With respect to syntactic category, the present progressive *-ing*, plural *-s* had higher accuracy than the third-person singular *-s*. The past tense *-ed* demonstrated high accuracy despite its low syntactic category score. Lastly, when it comes to semantic complexity, we could see semantically complex morphemes have lower accuracy than semantically simple morphemes. For instance, the third-person singular *-s* is semantically complex and it displayed the lowest production accuracy.

## Discussion

In this study, we examined the roles of L1 transfer, L2 exposure, and morphological salience in bilingual children's L2 English morpheme acquisition. Using language samples of naturalistic conversations from Paradis's (2005) corpus in CHILDES (MacWhinney, 2000), we analyzed the production of five English morphemes by Spanish-English bilingual children and Mandarin-English bilingual children learning English as an L2. Results revealed a significant effect of L1 transfer on the L2 morpheme accuracy such that SE bilinguals showed higher accuracy than ME bilinguals, especially for English plural *-s* and articles. In terms of L2 input quantity and quality, months of English exposure were shown to be a significant positive predictor for L2 morpheme production accuracy. While lexical diversity also contributes to morpheme accuracy for both groups of children, MLU seems to negatively affect ME bilingual children's production accuracy. In addition, our descriptive analyses of morphological salience factors indicate that, across both groups of children, perceptual salience, morphophonological regularity, syntactic category and semantic complexity may influence morpheme production accuracy to different degrees. Overall, the findings underlie the importance of L1 transfer, L2 exposure, morphological salience in bilingual children's L2 morpheme acquisition.

### The role of L1 transfer

The significant main effect of Group (SE vs. ME) demonstrates that the SE group on average had higher accuracy of English morpheme production compared to the ME group. This can be explained by different L1 morphological systems of the two bilingual groups. Spanish and Mandarin are two typologically different languages, where Spanish is morphologically rich/fusional while Chinese is morphologically isolating (Li and Thompson, 1981). Our results show that the richness of the L1 morphological system of early sequential bilingual children influences L2 morpheme production accuracy. This is in line with findings from previous studies such as Paradis (2011) which found that Mandarin- and Cantonese-speaking children had lower accuracy in L2 verbal morphology as compared to children whose L1s (e.g., Spanish) have tense and agreement marking.

In previous studies, a positive transfer has been often observed when a morpheme is shared in both L1 and L2 of the bilinguals. Thus, we predicted that ME bilinguals would produce the present progressive *-ing* with similar accuracy as the SE bilinguals, and that ME bilinguals would have lower accuracy in producing plural *-s*, the third-person singular *-s*, articles, and past tense *-ed*. Our results showed that the accuracy for progressive *-ing* were not significantly different between SE and ME bilinguals, as we predicted. In addition, we observed a significant group difference for English articles and plural *-s*, which could be attributed to the overlapping of these two morphemes between Spanish and English but not between Mandarin and English. Different from the prediction, the other two morphemes (i.e., past tense *-ed*; third-person singular *-s*) which overlap between Spanish and English did not reveal an advantage for SE bilinguals; ME bilinguals even showed descriptively higher TLU scores for past tense *-ed* than SE bilinguals. That is to say, the presence or absence of a morpheme in L1 is not sufficient to determine the morpheme production accuracy in the L2.

With respect to past tense *-ed*, during our analyses, we observed that SE bilingual children made more commission errors than ME bilingual children. For instance, SE bilingual children often oversupplied the past tense *-ed* in the production, producing the verb “*break*” as “*breaked*.” In contrast, ME bilinguals hardly made such commission errors but more omission errors. In other words, even though we did not observe a significant group difference in the past tense *-ed* production, the additional error analyses indeed support potential influence from L1 typology. In Spanish, past tense verbs are inflected for person, number, and formality, with distinct forms for each (e.g., *tú, usted, nosotros, ellos*). This added complexity might explain why SE bilinguals did not outperform ME bilinguals in past tense *-ed* production accuracy, as there is less overlap between Spanish and English in past tense morphology. However, we also believe that, in this case, SE bilingual children should find English past tense relatively easier, given that its rules are simpler than those of Spanish. Regarding the third-person singular *-s*, both groups demonstrated the lowest production accuracy among all five morphemes. This suggests that these bilingual children were still in the process of acquiring this morpheme, potentially leading to the limited variability in production accuracy and the lack of a significant group difference.

These findings revealed the interactive features of the two languages such that the morphophonological knowledge from the L1 can transfer to the process of L2 morpheme acquisition. Importantly, from a developmental acquisition perspective, our study suggests that learning L2 English morphemes may not be fully determined by whether there is an equivalent or overlapping morpheme in the L1 or not. The production accuracy is likely influenced by additional factors, such as L2 exposure and morphological salience, which will be discussed in the following sections.

### The role of L2 exposure

In this study, we examined input quantity and input quality to understand the effect of L2 exposure on bilingual children's L2 morpheme production accuracy. We observed that the months of English exposure (MOE) is a significant factor, indicating that more English exposure leads to higher accuracy for both groups of bilingual children. This finding aligns with the existing literature (e.g., Bedore et al., 2012; Hoff et al., 2012) which found that greater exposure to L2 English significantly enhanced young bilingual children's acquisition of vocabulary, grammatical complexity, and MLU3^3^ in English. As our study relied on existing corpus data, we were unable to analyze the effect of current language exposure but focused on cumulative language exposure. Despite this limitation, our findings align with previous research, supporting the facilitative role of language exposure in bilingual language development.

With regard to L2 input quality, our study examined the lexical diversity and morphological richness of the input. When both language groups were included in the model, we identified a significant main effect of lexical diversity in L2 morpheme production accuracy, namely, the more diverse vocabulary the input has, the more facilitative it will be for children's morpheme production accuracy. Prior studies have found that the lexical diversity of the teachers facilitated bilingual children's L2 vocabulary growth. Our study extends this line of research by demonstrating that exposure to more diverse vocabulary in the input was associated with higher accuracy in bilingual children's production of L2 morphemes. The effect of lexical diversity on morpheme production accuracy could be explained in several ways. When children are exposed to more diverse words in the input, they may encounter the use of the morphemes in different words and contexts, which helps decompose and process the morphemes. This diverse input provides them with opportunities to internalize the regularities of morpheme usages than hearing it with limited set of words. When they understand more vocabulary items, they can better notice and process the morphemes, rather than trying to understand the basic meaning of words.

In this study, we observed a negative effect of input's morphological richness, as measured by MLU. The negative effect of MLU on bilingual language development has been observed in some previous studies. For example, Bowers and Vasilyeva (2011) found that the MLU of teachers' input is negatively associated with bilingual children's vocabulary growth. Studies also have indicated that children would benefit from exposure to short and simple utterances at the early stage of learning (e.g., Berko-Gleason, 1977; Furrow et al., 1979). As Hoff (2006) suggested, higher morphological complexity may increase the cognitive processing load. It can be challenging for young children to process long and complex utterances, as a result of the developing working memory capacity. When children are not overwhelmed by multiple morphemes in the utterances, they might better decompose and process individual morphemes. The effect is only significant for ME bilingual children, rather than SE bilingual children, which could be explained by the L1 morphological typology. To illustrate, SE bilingual children's L1 is morphologically rich, so they are exposed to morphologically rich utterance more than ME bilinguals, which likely facilitates their processing of morphologically complex utterances in English. In contrast, ME bilingual children's L1 is less morphological complex, which may have put them at a disadvantage in processing morphological complex utterances. Note that while many existing research has focused on caregiver input, our results suggest that even brief, structured interactions during testing sessions can influence children's morpheme production accuracy. The effect of input quality indicates that bilingual children are sensitive to the linguistic environment and underscores the impact of diverse linguistic interactions on bilingual children's language development.

### The role of morphological salience

The current study exploratorily examined the influence of morphological salience on L2 English morpheme acquisition by bilingual children. Across both groups, semantic complexity of a morpheme seems to play a role, namely, morphemes that encode multiple meanings are generally more difficult to acquire than those with fewer meanings. A transparent relationship between form and meaning facilitates acquisition. Our findings (see Figures 1, 2) support this pattern. For instance, the third-person singular *-s*, which conveys meanings of person, number, and present tense, showed the lowest accuracy score, reflecting its semantic complexity.

Additionally, the syntactic category of a morpheme appears to be associated with its production accuracy. Zobl and Liceras (1994) suggest that the acquisition order of functors/morphemes can be explained by grouping morphemes into categories based on their syntactic roles. According to their theory, free lexical morphemes are acquired before bound functional ones. Note that our study focuses on bound functional morphemes and articles (i.e., free functional morphemes), which differ in syntactic categories based on the Functional Category Theory (Zobl and Liceras, 1994). In the coding (see Appendix B), the present progressive *-ing* and plural *-s* have the highest value, which are followed by articles, and then past tense -*ed* and third-person singular -*s*. In line with this, the results show that the present progressive *-ing* and plural *-s* exhibited higher accuracy, articles showed moderate accuracy, and the third-person singular *-s* demonstrated lower accuracy. However, for ME bilingual children, the past tense *-ed*, which is expected to show lower accuracy, instead demonstrated relatively high accuracy. This may be due to factors such as its frequency and its semantic transparency.

Regarding morphophonological regularity, no clear pattern was observed for either group. Only some morphemes seemed to be influenced by this factor. For instance, the third-person singular *-s* has three phonological alternations (/s/, /z/, /z/) and shares homophony with plural *-s*, resulting in relatively high morphophonological irregularity. These characteristics may make it more challenging to acquire compared to other morphemes. In contrast, morphemes such as the present progressive *-ing* and past tense *-ed*, which exhibit greater regularity, were produced more accurately by the children. Notably, while plural *-s* and articles are morphophonologically irregular, they had high accuracy scores, possibly because these morphemes had already been acquired by the bilingual children.

Our findings also revealed that perceptual salience may be associated with production accuracy for some but not all morphemes. For example, the accuracy of plural *-s* and past tense -*ed* do not seem to be affected by perceptual salience. This, however, aligns with arguments by O'Grady et al. (2017), who questioned the salience-based explanation for variability in inflectional morphemes. For example, O'Grady et al. argued that it is unlikely that learners struggle to perceive word-final -*s*, as studies show that typically developing children can distinguish *no* vs. *nose* and that young infants and children are sensitive to subject-verb agreement errors in preferential looking paradigms (e.g., Soderstrom et al., 2002).

Overall, among the salience factors, semantic complexity may play a role in L2 morpheme production accuracy, with more complex morphemes potentially being more challenging to acquire. Furthermore, the results regarding the syntactic category seem to in line with the prediction by the Functional Category Theory (Zobl and Liceras, 1994). Although this study takes a descriptive approach, the findings provide insights into how morphological salience factors could influence bilingual children's morpheme production.

## Conclusion

This study contributes to our understanding regarding the effects of L1 transfer, L2 exposure, and morphological salience on bilingual children's acquisition of L2 English morphemes. By examining two groups of bilingual children, we found that L1 transfer plays a critical role in shaping L2 English morpheme production. Our findings further highlight the importance of L2 exposure in explaining variability in bilinguals' L2 morpheme acquisition, particularly the amount of L2 exposure and the quality of interactive input, such as lexical diversity. Besides, our results suggest that difficulties in producing English morphemes may be associated with some morphological salience factors.

The current study is based on naturalistic conversational data from Paradis (2005) in CHILDES (MacWhinney, 2000). As the language samples are naturalistic and spontaneous, the number and types of verbs included in the transcripts could not be controlled. Some children may have produced certain morphemes (e.g., *-ing*) more accurately with familiar verbs (e.g., *eating, sleeping*), potentially leading to higher accuracy scores. Future research could address this limitation by employing experimental tasks (e.g., story retell) to elicit a broader range of morphemes with a variety of verbs. Moreover, the small sample size of bilingual children included in this study may limit the generalizability of the findings, and increasing the sample size would enhance the robustness of future analyses. While the descriptive approach used in this study provides some insights into how morphological salience factors might influence bilingual children's L2 English morpheme acquisition, our findings remain exploratory. Future research should build on these insights by employing experimental designs and inferential statistical analyses to rigorously test the role of morphological salience in L2 morpheme acquisition.

## Data Availability

Publicly available datasets were analyzed in this study. This data can be found here: https://childes.talkbank.org/access/Biling/Paradis.html.

## Footnotes

^1^These five morphemes were selected based on Goldschneider and DeKeyser (2001), which were commonly examined in the studies they meta-analyzed.

^2^We calculated MLUm from the first 200 intelligible utterances using MOR and MLU functions in CLAN (MacWhinney, 2000).

^3^MLU3 refers to the mean length of the three longest sentences.

## References

[B1] Bates D. Mächler M. Bolker B. Walker S. (2014). Fitting Linear Mixed-Effects Models Using lme4. Available online at: https://arxiv.org/abs/1406.5823 (accessed November 2023).

[B2] Bedore L. M. Peña E. D. Anaya J. B. Nieto R. Lugo-Neris M. J. Baron A. (2018). Understanding disorder within variation: production of English grammatical forms by English language learners. Lang. Speech Hearing Serv. Sch. 49, 277–291. 10.1044/2017_LSHSS-17-0027 29621806 PMC6105132

[B3] Bedore L. M. Peña E. D. Summers C. L. Boerger K. M. Resendiz M. D. Greene K.. (2012). The measure matters: language dominance profiles across measures in Spanish–English bilingual children. Bilingual. Lang. Cogn. 15, 616–629. 10.1017/S1366728912000090 23565049 PMC3615884

[B4] Berko-Gleason J. (1977). “Talking to children: some notes on feedback,” in Talking to Children: Language Input and Acquisition, eds. C. E. Snow & C. A. Ferguson (Cambridge: Cambridge University Press), 199–215.

[B5] Blom E. Paradis J. Duncan T. S. (2012). Effects of input properties, vocabulary size, and L1 on the development of third person singular –*s* in child L2 English. Lang. Learn. 62, 965–994. 10.1111/j.1467-9922.2012.00715.x

[B6] Blom E. Paradis J. Sorenson Duncan T. (2010). “The acquisition of 3SG-s by L2 children: domain-general or domain-specific learning?,” in Poster presented at the Child Language Seminar, City University, London, UK.

[B7] Bohman T. M. Bedore L. M. Peña E. D. Mendez-Perez A. Gillam R. B. (2010). What you hear and what you say: language performance in Spanish–English bilinguals. Int. J. Bilingual Educ. Bilingual. 13, 325–344. 10.1080/13670050903342019 21731899 PMC3128885

[B8] Bowers E. P. Vasilyeva M. (2011). The relation between teacher input and lexical growth of preschoolers. Appl. Psycholinguist. 32, 221–241. 10.1017/S0142716410000354

[B9] Brown R. (1973). A First Language: The Early Stages. London: George Allen & Unwin. 10.4159/harvard.9780674732469

[B10] Corbett G. G. (2000). Number. New York, NY: Cambridge University Press. 10.1017/CBO9781139164344

[B11] DeKeyser R. M. Alfi-Shabtay I. Ravid D. Shi M. (2018). “The role of salience in the acquisition of Hebrew as a second language,” in Salience in Second Language Acquisition (New York, NY: Routledge/Taylor & Francis), 131–146. 10.4324/9781315399027-7

[B12] Duanmu S. (2007). The Phonology of Standard Chinese. Oxford: Oxford University Press. 10.1093/oso/9780199215782.001.0001

[B13] Ellis N. C. (2018). “Salience in usage-based SLA,” in Saliency in Second Language Acquisition, eds. S. Gass, P. Spinner, & J. Behney (New York, NY: Routledge/Taylor & Francis), 21–40. 10.4324/9781315399027-2

[B14] Fenson L. Marchman V. A. Thal D. J. Dale P. S. Rez- nick J. S. Bates E. (2003). MacArthur-Bates Communicative Development Inventories: User's Guide and Technical Manual, 2nd Edn. Baltimore, MD: Paul H. Brookes.

[B15] Fisher C. Church B. A. Chambers K. E. Hall D. G. Waxman S. R. (2004). “Learning to identify spoken words,” in Weaving a Lexicon, eds. D. G. Hall and S. R. Waxman (Cambridge, MA: The MIT Press), 3–40. 10.7551/mitpress/7185.003.0004

[B16] Fox J. Weisberg S. (2018). An R Companion to Applied Regression. Thousand Oaks, CA: Sage Publications. 10.32614/CRAN.package.carData

[B17] Furrow D. Nelson K. Benedict H. (1979). Mothers' speech to children and syntactic development: some simple relationships. J. Child Lang. 6, 423–442. 10.1017/S0305000900002464 536408

[B18] Gass S. M. Spinner P. Behney J. (2017). “Salience in second language acquisition and related fields,” in Salience in Second Language Acquisition (New York, NY: Routledge/Taylor & Francis), 1–18. 10.4324/9781315399027

[B19] Gathercole V. C. M. (2002). “Command of the mass/count distinction in bilingual and monolingual children: an English morphosyntactic distinction,” in Language and Literacy in Bilingual Children, eds. D. K. Oller & R. E. Eilers (Clevedon, England: Multilingual Matters), 175–206. 10.21832/9781853595721-00937917319

[B20] Goldschneider J. M. DeKeyser R. M. (2001). Explaining the “natural order of L2 morpheme acquisition” in English: a meta-analysis of multiple determinants. Lang. Learn. 51, 1–50. 10.1111/1467-9922.00147

[B21] Green D. W. (1998). Mental control of the bilingual lexico–semantic system. Bilingual. Lang. Cogn. 1, 67–81. 10.1017/S1366728998000133

[B22] Guo L. Y. Eisenberg S. (2015). Sample length affects the reliability of language sample measures in 3-year-olds: evidence from parent-elicited conversational samples. Lang. Speech Hearing Serv. Sch. 46, 141–153. 10.1044/2015_LSHSS-14-0052 25615272 PMC4610271

[B23] Hartig F. (2024). DHARMa: Residual Diagnostics for Hierarchical (Multi-Level/Mixed) Regression Models. R package version 0.4.6. Available online at: https://CRAN.R-project.org/package=DHARMa (accessed January 1, 2025).

[B24] Hoff E. (2006). How social contexts support and shape language development. Dev. Rev. 26, 55–88. 10.1016/j.dr.2005.11.002

[B25] Hoff E. Core C. Place S. Rumiche R. Señor M. Parra M. (2012). Dual language exposure and early bilingual development. J. Child Lang. 39, 1–27. 10.1017/S0305000910000759 21418730 PMC4323282

[B26] Jackson-Maldonado D. Thal D. Fenson L. Marchman V. Newton T. Conboy B. (2003). MacArthur inventarios del Desarrollo de habilidades comunicativas (Inventarios): User's Guide and Technical Manual. Baltimore, MD: Brookes Publishing.

[B27] Jarvis S. Pavlenko A. (2008). Crosslinguistic Influence in Language and Cognition. New York, NY & London: Routledge. 10.4324/9780203935927

[B28] Jia G. (2003). The acquisition of the English plural morpheme by native Mandarin Chinese-speaking children. J. Speech Lang. Hearing Res. 46, 1297–1311. 10.1044/1092-4388(2003/101) 14700356

[B29] Jia G. Fuse A. (2007). Acquisition of English grammatical morphology by native Mandarin-speaking children and adolescents: age-related differences. J. Speech Lang. Hearing Res. 50, 1280–1299. 10.1044/1092-4388(2007/090) 17905912

[B30] Kuznetsova A. Brockhoff P. B. Christensen R. H. B. (2016). Package ‘lmerTest': R Package Ver-sion 2-0. Available online at: http://CRAN.R-project.org/package=lmerTest (accessed November 2023).

[B31] Laver J. (1994). Principles of Phonetics. Cambridge: Cambridge University Press. 10.1017/CBO9781139166621

[B32] Leow R. P. Martin A. (2017). “Enhancing the input to promote salience of the L2: a critical overview,” in Salience in Second Language Acquisition (New York, NY: Routledge/Taylor & Francis), 167–186. 10.4324/9781315399027-9

[B33] Li C. Thompson S. (1981). Mandarin Chinese: A Functional Reference Grammar. Berkeley, CA: University of California Press. 10.1525/9780520352858

[B34] Li Y. H. A. (1999). Plurality in a classifier language. J. East Asian Linguist. 8, 75–99. 10.1023/A:1008306431442

[B35] MacWhinney B. (2000). The CHILDES Project: Tools for Analyzing Talk. Transcription Format and Programs, Vol. 1. Psychology Press. 10.1162/coli.2000.26.4.657

[B36] Malvern D. Richards B. Chipere N. Durán P. (2004). Lexical Diversity and Language Development, 16–30. London: Palgrave Macmillan. 10.1057/9780230511804

[B37] McCarthy P. M. Jarvis S. (2007). vocd: a theoretical and empirical evaluation. Lang. Test. 24, 459–488. 10.1177/0265532207080767

[B38] McMillen S. Anaya J. B. Peña E. D. Bedore L. M. Barquin E. (2022). That's hard! Item difficulty and word characteristics for bilinguals with and without developmental language disorder. Int. J. Bilingual Educ. Bilingual. 25, 1838–1856. 10.1080/13670050.2020.1832039

[B39] Michael E. B. Gollan T. H. (2005). “Being and becoming bilingual: individual differences and consequences for language production,” in Handbook of Bilingualism: Psycholinguistic Approaches, eds. J. F. Kroll & A. M. B. de Groot (New York, NY: Oxford University Press), 389–407. 10.1093/oso/9780195151770.003.0022

[B40] Murakami A. (2014). Individual Variation and the Role of L1 in the L2 Development of English Grammatical Morphemes: Insights from Learner Corpora [Apollo - University of Cambridge Repository]. Cambridge: University of Cambridge. 10.17863/CAM.16509

[B41] Nicoladis E. (2012). Cross-linguistic influence in French–English bilingual children's possessive constructions. Bilingual. Lang. Cogn. 15, 320–328. 10.1017/S1366728911000101

[B42] Nicoladis E. Song J. Marentette P. (2012). Do young bilinguals acquire past tense morphology like monolinguals, only later? Evidence from French–English and Chinese–English bilinguals. Appl. Psycholinguist. 33, 457–479. 10.1017/S0142716411000439

[B43] Nicoladis E. Yang Y. Jiang Z. (2020). Why jumped is so difficult: tense/aspect marking in Mandarin–English bilingual children. J. Child Lang. 47, 1073–1083. 10.1017/S0305000920000082 32102710

[B44] Odlin T. (1989). Language Transfer, Vol. 27. Cambridge: Cambridge University Press. 10.1017/CBO9781139524537

[B45] O'Grady W. Kim K. Kim C. E. (2017). “The role of salience in linguistic development: a contrarian view,” in Salience in Second Language Acquisition (New York, NY & London: Routledge), 64–86. 10.4324/9781315399027-4

[B46] Paradis J. (2005). Grammatical morphology in children learning English as a second language: implications of similarities with specific language impairment. Lang. Speech Hearing Serv. Sch. 36, 172–187. 10.1044/0161-1461(2005/019) 16175882

[B47] Paradis J. (2010). Bilingual children's acquisition of english verb morphology: effects of language exposure, structure complexity, and task type. Lang. Learn. 60, 651–680. 10.1111/j.1467-9922.2010.00567.x

[B48] Paradis J. (2011). Individual differences in child English second language acquisition: comparing child-internal and child-external factors. Linguist. Approach. Bilingual. 1, 213–237. 10.1075/lab.1.3.01par33486653

[B49] Paradis J. (2023). Sources of individual differences in the dual language development of heritage bilinguals. J. Child Lang. 50, 793–817. 10.1017/S0305000922000708 36722256

[B50] Paradis J. Genesee F. (1996). Syntactic acquisition in bilingual children: autonomous or interdependent?. Stud. Second Lang. Acquisit. 18, 1–25. 10.1017/S0272263100014662

[B51] Place S. Hoff E. (2011). Properties of dual language exposure that influence 2-year-olds' bilingual proficiency. Child Dev. 82, 1834–1849. 10.1111/j.1467-8624.2011.01660.x 22004372 PMC4144198

[B52] Richtsmeier P. T. Gerken L. Goffman L. Hogan T. (2009). Statistical frequency in perception affects children's lexical production. Cognition 111, 372–377. 10.1016/j.cognition.2009.02.009 19338981 PMC2719879

[B53] Rowe M. L. Snow C. E. (2020). Analyzing input quality along three dimensions: interactive, linguistic, and conceptual. J. Child Lang. 47, 5–21. 10.1017/S0305000919000655 31668157

[B54] Schwartz M. Taha H. Assad H. Khamaisi F. Eviatar Z. (2016). The role of emergent bilingualism in the development of morphological awareness in Arabic and Hebrew. J. Speech Lang. Hear. Res. 59, 797–809. 10.1044/2016_JSLHR-L-14-0363 27561115

[B55] Soderstrom M. Wexler K. Jusczyk P. (2002). “English-learning toddlers' sensitivity to agreement morphology in receptive grammar,” in Proceedings of the 26th Annual Boston University Conference on Language Development, Vol. 2, eds. B. Skarabela, S. Fish, & A. Do (Somerville, MA: Cascadilla Press), 643–652.

[B56] Sun H. Yin B. Amsah N. F. B. B. O'brien B. A. (2018). Differential effects of internal and external factors in early bilingual vocabulary learning: the case of Singapore. Appl. Psycholinguist. 39, 383–411. 10.1017/S014271641700039X

[B57] Unsworth S. (2013). Assessing the role of current and *cumulative* exposure in simultaneous bilingual acquisition: the case of Dutch gender. Bilingual. Lang. Cogn. 16, 86–110. 10.1017/S1366728912000284

[B58] Unsworth S. (2016). “Quantity and quality of language input in bilingual language development,” in Lifespan Perspectives on Bilingualism, eds. E. Nicoladis & S. Montanari (Berlin: de Gruyter), 136–196. 10.1515/9783110341249-008

[B59] Whittle A. Lyster R. (2016). Focus on Italian verbal morphology in multilingual classes. Lang. Learn. 66, 31–59. 10.1111/lang.12131

[B60] Yip V. Matthews S. (2000). Syntactic transfer in a Cantonese–English bilingual child. Bilingual. Lang. Cogn. 3, 193–208. 10.1017/S136672890000033X

[B61] Zhang X. (2008). Chinese-men and Associative Plurals. Toronto Working Papers in Linguistics. Available online at: https://twpl.library.utoronto.ca/index.php/twpl/article/view/6156

[B62] Zobl H. Liceras J. (1994). Functional categories and acquisition orders. Lang. Learn. 44, 159–180. 10.1111/j.1467-1770.1994.tb01452.x

