# Supplementary material for: The roles of L1 transfer, L2 exposure, and morphological salience in bilingual children's L2 English morphological development
Source: Front Psychol. 2025 May 9;16:1566442. doi: 10.3389/fpsyg.2025.1566442 (PMC12098383; doi:10.3389/fpsyg.2025.1566442)
Supplement: Supplementary file 1 [file Data_Sheet_1.docx]

**Supplementary materials**

**Appendix A**

**Participant information from the CHILDES Database**

| **ID** | **L1** | **Biological Sex** | **AOA**  **(Months)** | **AOE**  **(Months)** | **Age at Testing**  **(Months)** | **MOE** | **MLUm** |
| --- | --- | --- | --- | --- | --- | --- | --- |
| CNDX1 | Mandarin | F | 73 | 73 | 81 | 8 | 2.958 |
| CNDX2 | Mandarin | F | 73 | 73 | 86 | 13 | 5.031 |
| CNDX3 | Mandarin | F | 73 | 73 | 93 | 20 | 5.41 |
| CNDX4 | Mandarin | F | 73 | 73 | 99 | 26 | 4.711 |
| DNNC1 | Mandarin | M | 53 | 55 | 64 | 9 | 2.928 |
| DNNC2 | Mandarin | M | 53 | 55 | 69 | 14 | 3.907 |
| DNNC3 | Mandarin | M | 53 | 55 | 75 | 20 | 3.939 |
| DNNS1 | Mandarin | M | 42 | 47 | 54 | 7 | 4.31 |
| DNNS3 | Mandarin | M | 42 | 47 | 67 | 20 | 4.347 |
| DNNS4 | Mandarin | M | 42 | 47 | 72 | 25 | 5.283 |
| JNNH1 | Mandarin | F | 53 | 53 | 71 | 18 | 2.792 |
| JNNH2 | Mandarin | F | 53 | 53 | 77 | 24 | 3.414 |
| JNNH3 | Mandarin | F | 53 | 53 | 83 | 30 | 5.337 |
| JNNH4 | Mandarin | F | 53 | 53 | 88 | 35 | 5.052 |
| JNNH5 | Mandarin | F | 53 | 53 | 95 | 42 | 4.175 |
| MRSS1 | Mandarin | F | 56 | 56 | 60 | 4 | 3.644 |
| MRSS2 | Mandarin | F | 56 | 56 | 66 | 10 | 3.465 |
| MRSS3 | Mandarin | F | 56 | 56 | 72 | 16 | 3.646 |
| MRSS4 | Mandarin | F | 56 | 56 | 78 | 22 | 4.144 |
| MRSS5 | Mandarin | F | 56 | 56 | 84 | 28 | 3.878 |
| TNYN1 | Mandarin | M | 68 | 70 | 77 | 7 | 3.673 |
| TNYN2 | Mandarin | M | 68 | 70 | 83 | 13 | 3.802 |
| TNYN3 | Mandarin | M | 68 | 70 | 89 | 19 | 3.824 |
| TNYN4 | Mandarin | M | 68 | 70 | 96 | 26 | 4.55 |
| TNYN5 | Mandarin | M | 68 | 70 | 103 | 33 | 4.12 |
| BRND1 | Spanish | M | 53 | 56 | 66 | 10 | 2.042 |
| BRND2 | Spanish | M | 53 | 56 | 72 | 16 | 2.887 |
| DVDC1 | Spanish | M | 67 | 67 | 75 | 8 | 3.594 |
| DVDC2 | Spanish | M | 67 | 67 | 82 | 15 | 5.122 |
| DVDC3 | Spanish | M | 67 | 67 | 87 | 20 | 5.392 |
| DVDC4 | Spanish | M | 67 | 67 | 93 | 26 | 4.273 |
| DVDC5 | Spanish | M | 67 | 67 | 99 | 32 | 4.424 |
| FLPP1 | Spanish | M | 45 | 58 | 68 | 10 | 3.51 |
| FLPP2 | Spanish | M | 45 | 58 | 75 | 17 | 4.566 |
| RNDL1 | Spanish | M | 88 | 89 | 94 | 5 | 2.929 |
| RNDL2 | Spanish | M | 88 | 89 | 100 | 11 | 2.66 |
| SBST1 | Spanish | M | 45 | 46 | 61 | 15 | 5 |
| SBST2 | Spanish | M | 45 | 46 | 67 | 21 | 4.95 |
| SBST3 | Spanish | M | 45 | 46 | 73 | 27 | 4.485 |
| SBST4 | Spanish | M | 45 | 46 | 80 | 34 | 3.747 |
| SBST5 | Spanish | M | 45 | 46 | 86 | 40 | 4.309 |
| SMNS1 | Spanish | M | 60 | 60 | 66 | 6 | 2.574 |
| SMNS2 | Spanish | M | 60 | 60 | 73 | 13 | 3.307 |
| SMNS3 | Spanish | M | 60 | 60 | 79 | 19 | 5.064 |
| SMNS4 | Spanish | M | 60 | 60 | 85 | 25 | 4.194 |
| SMNS5 | Spanish | M | 60 | 60 | 89 | 29 | 3.52 |

**Appendix B**

Salience scores for each morpheme

|  | Present progressive -*ing* | Plural -*s* | Possessive -*’s* | Articles | Past tense -*ed* | Third-person singular -*s* |
| --- | --- | --- | --- | --- | --- | --- |
| Number of phones (raw score) | 2 | 1.33 | 1.33 | 1.66 | 1.33 | 1.33 |
| Number of phones (*z* score) | 1.7996 | -.5959 | -.5959 | .5840 | -.5959 | -.5959 |
| Syllabic/nonsyllabic (raw score) | 1 | .33 | .33 | 1 | .33 | .33 |
| Syllabic/nonsyllabic (*z* score) | ﻿1.291 | –.6455 | –.6455 | 1.291 | –.6455 | –.6455 |
| Sonority (raw score) | ﻿11 | 5.33 | 5.33 | 10.33 | 3.66 | 5.33 |
| Sonority (z score) | ﻿1.3683 | –.4922 | –.4922 | 1.1485 | –1.0402 | –.4922 |
| Semantic complexity | 2 | 1 | 1 | 1 | 2 | 3 |
| Number of phonological alternations (raw score) | 2 | 3 | 3 | 4 | 3 | 3 |
| Number of phonological alternations (*z* score) | ﻿–1.5811 | 0 | 0 | 1.5811 | 0 | 0 |
| Homophony (raw score) | 1 | 2 | 2 | 1 | 1 | 2 |
| Homophony (*z* score) | ﻿–.9129 | ﻿.9129 | ﻿.9129 | ﻿–.9129 | ﻿–.9129 | .9129 |
| Syntactic category | 3 | 3 | 1 | 2 | 1 | 1 |
| Frequency | 160 | 147 | 71 | 552 | 44 | 89 |

Note: Reproduced Appendix D in Goldschneider & DeKeyser, (2001; p. 47).
